# Molecular docking analysis of arjunolic acid from *Terminalia arjuna* with a coronary artery disease target APOE4

**DOI:** 10.6026/97320630017949

**Published:** 2021-11-30

**Authors:** Lima Hazarika, Supriyo Sen, Jitesh Doshi

**Affiliations:** 1 Department of Biosciences, Assam Don Bosco University, Sonapur, 782402, Assam, India; 2 BioInsight Solutions Private Limited, Kharghar, Navi Mumbai,410210, Maharashtra, India

**Keywords:** Phyto compounds, apo lipoprotein, arjunolic acid, docking, coronary artery disease

## Abstract

Apo lipoprotein-E (APOE) encoded by APOE gene, is a plasma glycoprotein of 34.15 kDa and has a significant genetic association in coronary artery disease (CAD) progression. The silent epidemic of different cardiovascular diseases including CAD challenges novel therapeutic alternatives to prevent to treat chronic conditions of the disease and its associated complications. It is believed that natural phyto compounds and extracts have been a potential source of treating health conditions and have been practiced since several decades. The aim of the study is to identify phyto compounds having significant cardio protective activity targeting APOE4. Since protein-ligand interactions play a leading role in structure-based drug design, with the help of molecular docking, we selected 20 phyto chemicals present in different plants and investigated their binding affinity against targeted APOE isoforms. Among all selected phytoc ompounds, arjunolic acid, from Terminalia arjuna plant was found as promising candidate for developing therapeutic against APOE4 activated CAD. Findings from the present work could be further studied for clinical evaluations on human to adopt strategies and reduce the prevalence and mortality. Arjunolic acid derivatives can be used as a source of new medication or development of novel compounds in the treatment of CAD.

## Background:

Coronary artery disease (CAD) is a common heart condition that involves atherosclerotic plaque formation in the vessel lumen, leading to inadequate supply of blood and oxygen to the myocardium [1]. Apo lipoprotein-E (APOE) encoded by APOE gene, is a plasma glycoprotein of 34.15 kDa with 299-amino acids has revealed a pivotal role in the biological processes including CAD progression [2,3]. Studies discussed impairment of cholesterol efflux in CAD which has been linked to APOE4 accumulation in the endosomal compartments of the cells resulting in increased intracellular cholesterol production and atherosclerosis [4]. Significant amount of research is now focused on identifying new therapeutic alternatives from herbal and botanical origin to prevent and treat this disease. The WHO African Region and South-East Asia Region reported the highest percentage (>80%) of countries that utilizes traditional and complementary medicine (T&CM) clinically in hyper lipidaemia and ischemic heart disease [5]. The current era is well known for the plant-based medicinal framework due to their cost-effectiveness, easy availability, and known efficacy and reduced side effects[6].

Plant sterols, flavonoids, sulphur compounds, poly phenols, polysaccharides, antho cyanins and antioxidant natural products and their bioactive compounds influence plasma cholesterol, inhibit platelet aggregation, reduce blood pressure, improve lipid profile, mitigate oxidative stress, and inflammation indicating association to the atherosclerotic process in the cardiovascular system [7,8]. But characterization of these natural phyto compounds and their modes of action are not best established [7].Therefore, it is of interest to document the molecular docking analysis of arjunolic acid from Terminalia arjuna with a coronary artery disease target APOE4.

## Phyto compound Selection:

Traditional systems of medicines investigate a number of plants and found to have global potential for drug development. Indian Systems of Medicine (ISM) that includes medico-botanical surveys, cultivation of medicinal plants, phyto chemical studies, drug standardization, pharmacological and toxicological studies emphasize a few plants to have cardio protective nature, which were considered for the present study [9]. Studies on pharmacotherapy from medicinal plants used traditionally to treat coronary heart disease (CHD) suggest large number of phyto compounds with cardio protective potential and ethano-pharmacological properties that are valued as herbal medication and therapy to reduce the risk of cardiovascular disease but the underlying mechanism are still poorly understood [10]. Natural products and their derivatives represent more than 50% of drugs to contribute in complementary and alternative medicine (CAM) therapy [11]. Also, emerging scientific research and technological advances clearly indicate that natural phyto compounds will be a significant source of new medications [12]. The present study selected 20 different plant species documented to have some medicinal properties related to cardiovascular diseases (Table 1 - see PDF). The exact mechanism of cardio protection of the selected phyto compounds remains unclear. Therefore, it is important to understand the molecular interactions which will provide valuable insights in structure-based drug design, and eventually for developing broad spectrum drugs against coronary artery diseases.

## Methodology:

### Homology Modelling and Structural assessment:

The structure and the full-length sequence of APOE3 (PDB: 2L7B) was obtained from PDB database. The sequence for APOE4 was derived from the APOE3 sequence (PDB: 2L7B) by substituting the amino acids at position 130 (112 in mature protein) and position 176 (158 in mature protein), since the difference between the two isoforms lies at 112th position. BLAST analysis of APOE4 sequence was performed followed by multiple sequence alignment (MSA) with 2L7B and 1B68 and eventually to protein model building using Modeller 9.24. Homology modelling for an interactive visualization and analysis of molecular structures and related data, was performed, using UCSF Chimera interface [45]. The final model was selected based on the lower discrete optimized protein energy (DOPE) score which was -0.24 and was further was validated both on geometric and energetic scale using PROCHECK from PDBsum) and Discovery Studio. Model structure of APOE4 generated was further minimized using YASARA minimization server in presence of water as solvent to improve orientations [3].

### Target Protein Preparation:

The complete structure of APOE3 (PDB: 2L7B) and the modelled structure of APOE4 were used as template. The protein structures were prepared for docking, by protonating it at physiological pH. Hydrogen atoms were added with hydrogen bond network optimization. Charges for standard residues were calculated using Amber 14SB force field and for ligands Gasteiger charges were used [46].

### Phytocompound Selection:

The interactive chemical 3D structures of the phyto compounds were obtained from PubChem Database (Table 2 - see PDF) for the study [47].

### Ligand Preparation:

The 3D structures of above selected phyto compounds considered as ligands here, were obtained from PubChem database in SDF format and converted to PDB format using the tool Open Babel v3.1. The Gasteiger charges and rotatable bonds were assigned to the PDB ligands using Auto Dock Tools and the compound structures were energy minimized and considered for docking studies.

### Molecular Docking Simulations:

Molecular docking was used for predicting the binding affinities for the selected number of ligands. Docking was performed with AutoDock Vina v 1.1.2. [48]. Docking was performed to obtain a population of possible conformations and orientations for the ligand at the binding site. The best conformation was selected with the lowest docked energy and the interactions between selected phyto compounds and target receptor were visualized by using Discovery Studio.

## Results and Discussion:

The selected phyto compounds, known for its cardio protective potential in heart failure, ischemic, cardiomyopathy, atherosclerosis and cholesterol metabolism in heart conditions, were selected to computationally evaluate their candidature as prospective APOE4 modulators. Among all the phyto compounds, Oil of Garlic (6850738) and Fennel oil (6850740) did not produce any significant binding pose against APOE isoforms. Results obtained on docking selected phyto compounds with APOE4 compared to APOE3, is shown in Table 2(see PDF).

## Functional analysis:

Comparison between the Binding affinities:

The data of binding affinities of selected phyto compounds with target proteins is presented in Table 2(see PDF). Evident from the docking study, it was found that digitoxin/ digitoxoside (CID: 441207) exhibited highest binding affinity with APOE4 (-9.0 kcal/mol) among the selected compounds. It showed even better affinity towards APOE3 (-9.3 kcal/mol). In spite of its high affinity and high efficacy, the focus was to find decent affinity towards APOE4 as target. Arjunolic acid (CID: 73641) presented a strong and a tight binding affinity of -8.7 kcal/mol with APOE4 whereas it showed -7.6 kcal/mol with APOE3, with 1.1 kcal/mol difference in binding energy. In case of E-Guggulsterone (CID: 6439929) the difference in binding energy with APOE4 and APOE3 was -0.9 kcal/mol and that found in Fenugreek (CID: 91864538) was -1.1 kcal/mol. Docking results of Arjunolic acid with targeted APOE proteins had a good binding affinity and better binding mode and this can be considered for further research for therapeutics of coronary artery disease.

## Interaction analysis:

Binding affinities are influenced by non-covalent intermolecular interactions such as hydrogen bonding, electrostatic interactions, hydrophobic and van der Waals forces between the two molecules. The present study found Alanine (178), Glutamine (35) and Arginine (292) of APOE4 interacted with hydrogen bonds of Arjunolic acid (PubChem ID: 73641) but Aspartic acid (271) and Lysine (157) were involved in case of APOE3. Hydrogen bond interaction, and hydrophobic interactions of the phyto compounds with both the receptors were analysed using Discovery Studio and are summarized in Figure 2(see PDF). While Arjunolic acid exhibited strong H-bond interaction with the APOE variants, it is observed that the compound shared four stable H-bonds with APOE4 but shares two H-bond with APOE3. There are no unfavourable bonds between the protein and the ligand in both the cases. Compared to this, E-Guggulsterone (CID: 6439929) exhibited van der waals interaction by Leucine (232) and Pi-Sigma bonding by Tryptophan (228) with APOE4 receptor; while C-H bonds by Arginine (264) and alkyl bonds by Alanine (160) with APOE3 receptor. Similarly, Fenugreek (CID: 91864538) exhibited unfavourable bonding with both APOE4 and APOE3. This projects Arjunolic acid as a candidate compound to be used as therapeutic for APOE4 activated CAD. The docking scores and the protein-ligand interactions suggest that arjunolic acid has the ability to efficiently bind to target protein involved in coronary artery disease involving APOE4.

## Analysis of binding affinity:

Using the structural information of both the protein and the ligands, accurate prediction of binding affinities by KDEEP was made which is based on 3D-convolutional neural networks [49]. Estimation of binding affinities using KDEEP also showed a very strong affinity of arjunolic acid towards APOE4 than APOE3 and the calculated ligand efficiency for APOE4 was -0.45 kcal/mol and that for APOE3 was -0.36 kcal/mol. The (Kd and binding free energy) for the docked protein-ligand complexes was evaluated and the standard free-energy change (std. ΔG) for APOE4 was -15.58 kcal/mol and that for APOE3 was -12.54 kcal/mol, which confirms favourable and tight binding affinity between APOE4 and arjunolic acid (Tables S1 o S3 - see PDF)).

Analysis of the binding affinity was also performed for the phyto compounds viz, E-Guggulsterone (PubChem ID: 6439929) and Fenugreek (PubChem ID: 91864538). The calculated ligand efficiency of E-Guggulsterone for APOE4 was -0.21 kcal/mol and that for APOE3 was -0.64 kcal/mol; the same was calculated for Fenugreek, which was -0.15 kcal/mol for APOE4 and -0.19 kcal/mol for APOE3. It can be predicted that comparatively, arjunolic acid seemed proficient to have stable binding affinity with APOE4 and therefore, could be further investigated with in vivo validations.

Research has now directed towards targeting the natural phyto compounds and plants extract that represents potential lead for novel drugs for CAD [50]. Figure 1(see PDF) is a graphical representation of the selected natural phyto compounds from plants with potential benefits on the cardiovascular system including coronary artery disease. Table 1(see PDF) shows selected phyto compounds present in various medicinal plants and their therapeutic benefits in cardiovascular disease that can be considered and explored more in "postgenomic" era for pharmacological effects in human subjects.

Molecular docking study reflects different binding affinities in the two APOE isoforms with selected phyto compounds (Table 2 - see PDF). The difference in binding affinities (functionality) of APOE isoforms owes to the structural difference of cystine to arginine amino acid at 112th position in the APOE protein, that influences the LDL receptor binding site of the protein [2]. The structural differences at 112th position between the APOE isoforms might affect the protein behaviour leading to their variability association with the lipoprotein particle classes in the plasma and hence results in different functional characteristics [51].

In the study, the binding conformation between arjunolic acid and APOE isoforms is represented by intermolecular H-bond interaction (Figure 2 and 3 - see PDF). Binding conformation predicted by interactions are influenced by non-covalent intermolecular interactions such as hydrogen bonding, electrostatic interactions, hydrophobic and Van der Waals forces between the two molecules [52]. It is known that hydrogen bonds trengthens diverse cellular functions by regulating molecular interactions such as protein folding, protein-ligand interactions and catalysis, thereby, greatly enhance protein-drug binding affinity [53]. The four H-bond pairing demonstrates strong affinity between arjunolic acid with APOE4compared to that in APOE3 (Figure 2 - see PDF). Additionally, binding affinity analysis by KDEEP, suggests std. ΔG for arjunolic acid and APOE4 (-15.58 kcal/mol) indicates spontaneous favourable reaction and more stable affinity compared to that with APOE3 (-12.54 kcal/mol). It is believed that a change of around 1.4 kcal/mol in the free energy corresponds to a tenfold change in the free energy of binding [54] and the differing thermodynamic parameters i.e., negative Gibbs' free binding energy in APOE isoforms complexed with arjunolic acid suggests process of spontaneity and stability.

Studies investigated effective drugs for CAD, various compounds and substructures such as Statins, Platelet Aggregation Inhibitors, Peripheral Vasodilators, Factor-Xa inhibitors, Aspirin, Cholesterol Absorption Inhibitors, Calcium channel blockers, Bile acid sequestrates, Beta Blockers (Cardio selective), Beta Blockers (Non-Cardio selective), Angiotensin Converting Enzyme Inhibitors, anti lipidemic activity, Bioactive Molecules from ChEMBL, Stabilizers, utilizing molecular docking [3]. The present study found arjunolic acid to exhibit better binding affinity to APOE4 compared to the previously studied existing drugs and ligand modulators. It can be said that with respect to the docking studies on different chemical stabilizers, modulators and existing drugs, Arjunolic acid exhibited enhanced binding affinity (-8.7 kcal/mol) with APOE4.

Studies onphytochemical constituents of various parts of T. arjunaare used as cardiotonic in heart failure, ischemic, cardio myopathy, atherosclerosis and believed to reduce the risk of coronary artery disease (CAD) [14, [55,56]. Observational studies already reported the therapeutic benefits of the plant on systolic and diastolic functions improving the heart functional capacity in patients of dilated cardiomyopathy [57]. The present molecular docking study shows strong binding affinity of APOE4 with arjunolic acid can have protective or preventive effects on CAD. To establish and understand the exact mechanism of this interaction, further wet lab experimental studies are warranted. To understand the dynamic structure for binding analysis and understand the factors driving protein characteristics, molecular dynamics (MD) simulation approaches for the APOE isoforms in CAD should also be adopted. The present work on molecular docking suggests the vital need for further clinical exploration in large number of subjects on phytocompounds especially arjunolic acid from T. arjuna. Future research ought to investigate the degree of synergistic or the antagonistic impacts of various bioactive plant components through clinical studies and affirm the clinical efficacy and safety of the compounds.

## Conclusion:

The present work is an attempt to computationally screen and identify phyto compounds, known to have cardio protective nature, from different plant species which can bind to APOE4 receptor involved in CAD. We document the molecular docking analysis of arjunolic acid from Terminalia arjuna with a coronary artery disease target APOE4 for further consideration in drug discovery and development.

## Funding:

This research received no specific grant from any funding agency in the public, commercial, or not-for-profit sectors.

## Servers used with URL:

PDB database (https://www.rcsb.org/) Modeller 9.24 (https://salilab.org/modeller/) UCSF Chimera interface (https://www.cgl.ucsf.edu/chimera/) PROCHECK from PDBsum(https://www.ebi.ac.uk/thornton-srv/software/PROCHECK/) Discovery Studio (https://discover.3ds.com/discovery-studio-visualizer-download)YASARA minimization server (http://www.yasara.org/)
